# Fully Bayesian hierarchical modelling in two stages, with application to meta-analysis

**DOI:** 10.1111/rssc.12007

**Published:** 2013-08

**Authors:** David Lunn, Jessica Barrett, Michael Sweeting, Simon Thompson

**Affiliations:** Medical Research Council Biostatistics UnitCambridge, UK

**Keywords:** Abdominal aortic aneurysm, Bayesian hierarchical modelling, BUGS, Markov chain Monte Carlo methods, Random-effects meta-analysis

## Abstract

Meta-analysis is often undertaken in two stages, with each study analysed separately in stage 1 and estimates combined across studies in stage 2. The study-specific estimates are assumed to arise from normal distributions with known variances equal to their corresponding estimates. In contrast, a one-stage analysis estimates all parameters simultaneously. A *Bayesian* one-stage approach offers additional advantages, such as the acknowledgement of uncertainty in all parameters and greater flexibility. However, there are situations when a two-stage strategy is compelling, e.g. when study-specific analyses are complex and/or time consuming. We present a novel method for fitting the full Bayesian model in two stages, hence benefiting from its advantages while retaining the convenience and flexibility of a two-stage approach. Using Markov chain Monte Carlo methods, posteriors for the parameters of interest are derived separately for each study. These are then used as proposal distributions in a computationally efficient second stage. We illustrate these ideas on a small binomial data set; we also analyse motivating data on the growth and rupture of abdominal aortic aneurysms. The two-stage Bayesian approach closely reproduces a one-stage analysis when it can be undertaken, but can also be easily carried out when a one-stage approach is difficult or impossible.

## 1. Introduction

### 1.1. Standard methods of meta-analysis and their limitations

Meta-analysis is often undertaken in two stages, even when individual participant data are available. At the first stage, each study is analysed to provide an estimate of the parameter of interest, together with its standard error. At the second stage, the estimates are combined across studies; in a random-effects meta-analysis, potential heterogeneity between the study-specific parameters is permitted (Higgins *et al.*, 2009).

Writing *x*_*i*_ as the estimate of parameter *θ*_*i*_ in study *i*, with the standard error denoted by *s*_*i*_, the usual two-stage random-effects meta-analysis model is

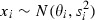
 within each study *i*, and

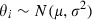
 across studies *i*=1,…,*N*, where *σ*^2^ is the between-study heterogeneity variance. In practice, 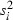 is estimated but then assumed to be without error in the above model, and normal distributions are assumed at both the first and the second stages. Although estimation of the overall parameters *μ* and *σ*^2^ can be by maximum likelihood or restricted maximum likelihood, a (non-iterative) moment estimator of *σ*^2^ is most often used in practice. The inference about *μ* is usually made by using an asymptotic normal approximation (i.e. asymptotic with respect to the number of studies), assuming that *σ*^2^ is fixed and known.

A Bayesian version of this model, in which study-specific and overall parameters are estimated simultaneously, can be implemented straightforwardly by using Markov chain Monte Carlo (MCMC) methods (Gelfand and Smith, 1990; Metropolis *et al.*, 1953; Hastings, 1970). This has several advantages: for example, uncertainty on all parameters, including *σ*^2^, is acknowledged simultaneously, prior information may be incorporated (e.g. Smith *et al.* (1995)), a credible interval for *μ* can simply be taken from the quantiles of its estimated posterior distribution, with no asymptotic normal approximation needed, and although the normality assumption for the between-study model is usually retained, a more flexible distribution could be used in principle (Lee and Thompson, 2008).

The focus of this paper, however, is on exploiting individual participant data, where available, to avoid the need for two potentially limiting assumptions in the above model:

- that the study-specific estimates are normally distributed;
- that the associated uncertainties (variances) are known.

The former may be inappropriate for studies with relatively sparse data, or when the parameters of interest are unconventional. The latter is circumvented with individual participant data because the full uncertainty regarding study-specific parameters is naturally propagated into the between-study model, and feedback is allowed from the between-study model to the estimation of study-specific parameters. For simple data structures, a non-Bayesian analysis can be achieved by using linear mixed models for continuous outcomes, or generalized linear mixed models for binary outcomes. The inference about *μ*, however, is again usually made by using an asymptotic normal approximation, assuming that *σ*^2^ is fixed and known (Higgins *et al.*, 2001). Alternatively, a Bayesian analysis can be implemented using MCMC sampling. In addition to the advantages that were outlined above, MCMC methods can be used when the study-specific data structures are complex.

Meta-analyses that make use of individual participant data are currently less common than their aggregate data counterparts, but their application is on the rise, especially in medicine (Riley *et al.*, 2010; Thompson *et al.*, 2010). Riley *et al.* (2010) presented a graphical summary of the trend over time, which shows around 50 such analyses per year being published by 2008. The Cochrane library now contains over 70 such analyses.

### 1.2. Two-stage Bayesian methods

This paper focuses on analysis of the *full hierarchical model*, in which the individual participant data are used to estimate study-specific and overall parameters simultaneously. A two-stage strategy, in which study-specific parameters are estimated separately in stage 1, is very attractive in several situations, however. In this paper we propose a novel method for fitting the full hier archical model in two stages. The idea is to fit a model to each study’s data independently in stage 1. The resulting study-specific posterior distributions are then used as proposal distributions for the study-specific parameters in stage 2, where those parameters are assumed to arise from a common population distribution (with unknown mean and variance, say). We describe the approach in detail in Section 3 but outline here several scenarios in which it may be useful.

- *When study-specific analyses are complex and/or time consuming*: study-specific data structures may be complex, requiring study level hierarchical models, with complex and/or non-linear regressions, say. Different studies may require different models, with different parameterizations possibly (although there must, by definition, be common parameters of interest across studies). It may thus be cumbersome to assemble computer code for analysing all studies simultaneously. If study-specific analyses are time consuming then a simultaneous analysis may be prohibitively so. A two-stage approach allows the analyst to consider the studies one by one, tailoring each analysis to the individual study and directly addressing any study-specific issues that may arise, such as convergence difficulties in an MCMC simulation—if, for example, posterior correlations between parameters are large for some studies, necessitating *long* simulations, there is no need to apply the same ‘run length’ to all studies. In fact, it is quite natural initially to explore the studies separately anyway, to identify appropriate models, to ensure that study-specific inferences make sense and to establish a model for linking the studies together.
- *When there are several models or parameters of interest to consider*: in cases where we wish to examine any relationships that may exist between the study-specific parameters and study level covariates, a two-stage approach allows these to be explored efficiently, without having to analyse the study-specific data repeatedly. Similarly, if there are several models to be entertained for fitting the study-specific data, these can be explored without having to fit the full hierarchical model. The effect of study-specific assumptions on overall inferences can then be readily explored in stage 2. Sometimes there may be multiple parameters of interest, such as predictive quantities for a range of prespecified conditions. Using MCMC methods for our study-specific analyses means that we can obtain study-specific inferences for *any* parameterization of interest simply by transforming the MCMC output. Overall inferences are then simply a matter of running a computationally efficient second stage for each parameter set of interest.
- *When the parameters of interest are complex functions of the ‘natural’ parameters*: in such cases it may be cumbersome to express the likelihood in terms of the parameters of interest, which is a fundamental requirement for a one-stage analysis. Sometimes this may even be impossible, because we cannot invert, algebraically, the relationship between parameters of interest and natural parameters (those that the likelihood is naturally expressed in terms of), although this inversion could, in principle, be performed numerically. Either way, a one-stage analysis is then problematic. Our proposed two-stage method offers a convenient way around this problem, exploiting again the fact that study-specific inferences for any parameterization of interest can be obtained by transforming appropriate MCMC output.

The motivating data that we consider below exemplify all the above three scenarios. They require complex, study level hierarchical models, and we are interested in many complex functions of the natural parameters. We would not realistically have been able to perform such an analysis without the developed two-stage methodology.

Although the above motivation for our work is in terms of meta-analysis, it is likely that two-stage or multistage Bayesian methods would have a range of other applications that could be explored. For example, in population pharmacokinetics, a potentially complex non-linear regression is fitted to repeated measurements from each of a number of individuals (e.g. Lunn *et al.* (2002)). Interindividual variability among the resulting parameters can sometimes be partially explained by various individual level covariates, providing scope for individualized dosage regimens in the target population. A two-stage approach could expedite the search for important covariates.

This paper is aimed at both methodological and applied statisticians. The methods are described in sufficient detail that they may be straightforwardly implemented in a low level (or high level, e.g. R (Ihaka and Gentlemen, 1996)) language of choice, or extended to other application areas. For readers who are less interested in the methodological detail, an implementation of the approach within the BUGS software (Lunn *et al.*, 2000, 2009) has been developed. The structure of the paper is as follows. In Section 2 we describe illustrative data on the effect of diuretics on risk of pre-eclampsia during pregnancy, as well as motivating data relating to the growth and rupture of abdominal aortic aneurysms (AAAs) (Sweeting and Thompson, 2011). In Section 3 we describe, in detail, the two-stage fully Bayesian approach, highlighting both its extensions and its limitations. Section 4 presents analyses of the data described in Section 2, whereas Section 5 contains a concluding discussion. Details regarding the implementation of our method in BUGS are provided in an on-line appendix.

## 2. Examples

### 2.1. Pre-eclampsia data

We illustrate our method by using a simple data set examining the effect of taking diuretics on the risk of pre-eclampsia during pregnancy. This data set was originally presented in Collins *et al.* (1985) and has been reanalysed in several more recent publications, including Thompson and Pocock (1991). It comprises the number of cases of pre-eclampsia recorded in both treatment and control groups in nine randomized trials published during the years 1962–1980. The data are given in the on-line appendix A.4.

### 2.2. Abdominal aortic aneurysms data

Our motivating problem concerns 14 studies providing longitudinal measurements of AAA diameter, made by ultrasound or computed tomography scan, together with the occurrence of clinical events, in particular rupture, surgery and death (RESCAN Collaborators, 2012). A joint model has been previously proposed to associate the size and growth of the aneurysm with the risk of AAA rupture (Sweeting and Thompson, 2011). A two-stage approach is particularly attractive in this setting for three reasons:

- analysis of the individual studies is complex and time consuming;
- there are many parameters of interest, representing predictions across a wide range of conditions;
- many of the parameters of interest are complex functions of the natural parameters.

The focus of the analysis is on growth and rupture rates for the ‘small’ AAA diameter range, 30–54 mm, where individuals are usually monitored without surgical intervention. Our aim is to quantify both the probability of rupture and the probability of crossing the surgical intervention threshold (55 mm) before the next scan, to inform appropriate intervals between monitoring scans.

The size of the studies ranges from 224 to 2227 patients, with a mean of 899 patients per study. An average of 5.9 AAA diameter measurements are available per patient. The average study follow-up is 4.2 years, although this ranges from 0.9 to 8.5 years between studies. The number of small AAA ruptures that are observed during follow-up ranges from 1 to 60, giving rise to a large range of (crude) rupture rates varying from 0.7 to 11 per 1000 person-years. The full data are not publicly available. However, an example data set, comprising observations on 100 randomly chosen individuals from a single study, is available in the on-line supplement to Sweeting and Thompson (2011).

## 3. Methods

### 3.1. Generic model

We describe the generic problem of interest as follows. Suppose that we have *N* studies indexed by *i*=1,…,*N*. Let *y*_*i*_ denote the data from study *i*. At the first level of our hierarchical model we define the study-specific likelihoods by specifying the sampling distribution of *y*_*i*_ conditional on ‘natural’ study-specific parameters *φ*_*i*_ and any ‘nuisance’ parameters *λ*_*i*_:

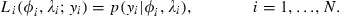


For example, suppose that our studies measure the number of patients responding positively to a particular treatment. In this case we would have

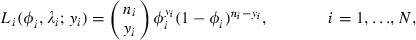
 where *n*_*i*_ is the total number of patients in study *i*, *φ*_*i*_ represents the underlying success rate for study population *i*, and in this case *λ*_*i*_ is empty.

We include ‘nuisance’ parameters in our formulation of the model merely to illustrate how certain parameters may be ignored in the second stage of our method, as long as the requisite assumptions of independence (see below) are appropriate. If these assumptions are not appropriate then *λ*_*i*_ can always be included within *φ*_*i*_. For example, if the response is normally distributed, we may choose whether to model the response standard deviation as a nuisance parameter or to include it within *φ*_*i*_ along with the mean.

Often the ‘parameters of interest’, about which we wish to make overall inferences, will be functions of the ‘natural’ parameters *φ*_*i*_. Denoting the parameters of interest in study *i* by *θ*_*i*_, we express this relationship as

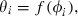
 or

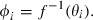


The 1:1 mapping *f* is typically chosen so that the *θ*_*i*_s are defined on the whole real line. For example, we may choose 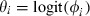 in the binomial example that was outlined above (hence *θ*_*i*_ would be the log-odds of success in study *i*). This is because we shall usually wish to assume, for interpretability and to facilitate covariate modelling, that the *θ*_*i*_s arise from a common normal distribution with unknown mean *μ* and covariance Σ,

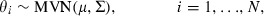
 although any appropriate population distribution could be used in principle (e.g. Lee and Thompson (2008). Hence the *θ*_*i*_s are typically assumed, at the second level of our hierarchical model, to be *conditionally* independent, given *μ* and Σ. (However, conditional independence is not a *necessary* assumption here.) In contrast, any nuisance parameters are assumed independent of the *θ*_*i*_s and *marginally* independent between studies: 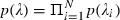, where *λ* denotes the set of all *λ*_*i*_s. As noted above, if this is not an appropriate assumption for any of the parameters that are included in *λ*_*i*_ then those parameters can simply be included within *φ*_*i*_ (and *θ*_*i*_) instead.

In some situations the parameters of interest *θ*_*i*_ may be complex functions of the natural parameters *φ*_*i*_ and it may be cumbersome, or not possible, to invert this relationship (to obtain *f*^−1^). Our motivating problem includes an example of such a situation, where one parameter of interest is the probability that an aneurysm will rupture in a given time. In such cases it may be difficult, or even impossible (e.g. 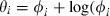)), to express the study-specific likelihoods, 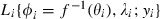, in terms of the parameters of interest algebraically, thus hindering analysis by any standard means. The two-stage approach that is proposed herein offers a convenient way around this problem.

At the third level of our model we assign prior distributions to *μ* and Σ, e.g. multivariate normal and inverse Wishart respectively. The joint posterior distribution is then given by

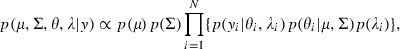
(1) where *y* and *θ* denote the collections of all *y*_*i*_s and all *θ*_*i*_s respectively, and

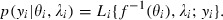


### 3.2. Inference

Our proposed method enables inferences on the full hierarchical model (1) but is performed in two stages, as described below.

#### 3.2.1. Stage 1

We first analyse all studies independently, to obtain a sample of size *B*_*i*_, *i*=1,…,*N*, from the joint posterior distribution of each *θ*_*i*_ and *λ*_*i*_, conditional on *y*_*i*_ alone, i.e.

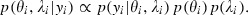
(2)

We denote the resulting samples by 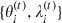, *t*=1,…,*B*_*i*_, *i*=1,…,*N*. These may be obtained either by MCMC simulation (using BUGS, say) from expression (2) directly, or by transforming the samples from MCMC simulation under an alternative parameterization, e.g. 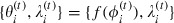 in the case where MCMC sampling under the model parameterized by *φ*_*i*_ has been performed. In the former case independent prior distributions are specified for each *θ*_*i*_ directly; in the latter case these are *implied* by independent priors specified for each *φ*_*i*_, say.

#### 3.2.2. Stage 2

Stage 2 comprises a Gibbs sampling scheme (Gelfand and Smith, 1990) in which we iteratively sample from the joint posterior distribution of *μ*, Σ, *θ* and *λ* under the full hierarchical model (1). At each iteration we cycle through the full conditional distributions for *μ*, Σ and then each *θ*_*i*_ and *λ*_*i*_ jointly. From distribution (1) these are given by

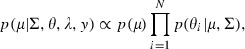
(3)

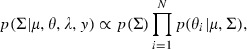
(4)

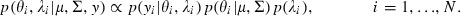
(5) Distributions (3) and (4) will typically be available in closed form, e.g. if multivariate normal and inverse Wishart priors are specified for *μ* and Σ respectively, and we can sample from them directly by using standard algorithms (e.g. Ripley (1987)). Otherwise, there are numerous alternative methods (e.g. Metropolis *et al.* (1953), Hastings (1970), Neal (1997) and Gilks and Wild (1992)) that can be employed.

For updating each *θ*_*i*_ and *λ*_*i*_, it makes intuitive sense to use the stage 1 posterior as a proposal distribution within a Metropolis–Hastings step (Metropolis *et al.*, 1953; Hastings, 1970). For a random variable *w* with density *p*(*w*), we can generate a *dependent* sample from *p*(*w*), under the Metropolis–Hastings algorithm, by constructing the Markov chain

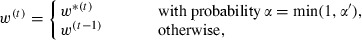

*t*=1,2,…, where w^*(*t*)^ is a ‘candidate value’ drawn from a proposal distribution *q*(*w*) that provides a reasonable approximation to the ‘target’ density *p*(*w*) but is easy to sample from. The ‘acceptance probability’ for the candidate value is a function of both the candidate w^*(*t*)^ and the preceding value *w*^(*t*−1)^:

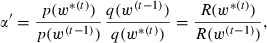
 where *R*(*x*) denotes the target-to-proposal density ratio *p*(*x*)/*q*(*x*). In the case of *θ*_*i*_ and *λ*_*i*_, proposing candidate values simply entails choosing stage 1 samples at random, by picking an index *c*_*it*_ uniformly from {1,…,*B*_*i*_} at each iteration *t*. Then

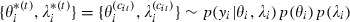
(6) from distribution (2). From expressions (5) and (6) the target-to-proposal ratio is therefore

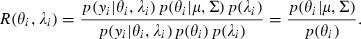
(7)

If we suppose that the stage 1 prior for *θ*_*i*_ is effectively uniform, and hence that stage 1 inferences or samples are based solely on the likelihood, then 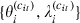 is accepted with probability min(1,*α*^′^), where

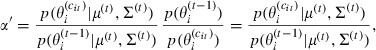
(8) where *μ*^(*t*)^ and Σ^(*t*)^ denote the values of *μ* and Σ respectively at iteration *t* of the Gibbs–Metropolis scheme. Note that the acceptance probability does not depend on *λ*_*i*_, and so the *λ*_*i*_s can actually be ignored in stage 2.

The cancellation of likelihood terms in equation (7) means that stage 2 can be performed very quickly, providing scope for rapid exploration of different level 2 models, such as in covariate selection. A further consequence of this cancellation is that we do not need to re-express the likelihood when reparameterizing the model, say for a different set of parameters of interest, since we can obtain stage 1 samples for any parameterization of interest by transforming those obtained for the original parameterization. Hence it is straightforward to make inferences about complex functions of the natural parameters, whose study-specific likelihoods may be cumbersome, or even impossible, to express algebraically. It is also straightforward to handle situations in which there are *many* parameters of interest, perhaps predictions over a range of conditions, since we can simply keep transforming the original stage 1 sample and rerunning stage 2, rather than repeatedly redefining the likelihood and performing a full analysis. We illustrate both of these situations in Section 4.2. A flat prior for one set of parameters does not necessarily imply a flat prior for some transformation of those parameters. Hence, when exploiting the option to transform our stage 1 output, we must be careful to check that the implied priors for *θ*_*i*_, *i*=1,…,*N*, are effectively flat, within the range of values that are supported by the stage 1 posterior. This is easy to verify following stage 1, however—see the on-line appendix A.3. If the implied priors for any derived parameters are not relatively flat, and we cannot parameterize stage 1 in terms of those parameters, or if flat priors are not considered appropriate (on subject matter grounds, say), then our method is still applicable as long as we retain the 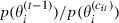 term in equation (8), although we have yet to explore whether this could lead to unacceptably low acceptance rates for some situations. Stage 2 analyses may be performed for one parameter at a time, for a complete set or for subsets of interest. Parameters that are excluded from a given analysis are essentially assumed to be nuisance parameters. Convergence of the MCMC simulation in stage 2 can be assessed by standard means (Cowles and Carlin, 1996; Mengersen *et al.*, 1999).

### 3.3. Hierarchical models with more than three levels

The method extends straightforwardly to hierarchical models with more than three levels. Suppose that we have a hierarchical model with *M* levels and we wish to split the analysis at level m^*^, so that independent posteriors for the parameters of interest at level m^*^ are obtained in stage 1, and these are then used as proposal distributions for those parameters in stage 2. The method proposed will work if distributional assumptions for nuisance parameters at level m^*^ and all parameters or data in levels *m*=1,…,m^*^−1 are identical in both the stage 1 and the full hierarchical models. This ensures that the cancellation in equation (7) will occur. All parameters in levels *m*=m^*^+1,…,*M* can be updated by standard Gibbs steps in stage 2. This extension allows for situations in which repeated measurements have been made on each individual within each study, say, and the *θ*_*i*_s above represent study level summaries of individual-specific parameters. Indeed, our motivating data set regarding AAAs has such a structure.

### 3.4. Specific models

#### 3.4.1. Pre-eclampsia model

Let the number of cases of pre-eclampsia in the control and treatment groups of study *i* be denoted by 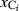 and 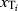 respectively. Further, denote the corresponding underlying pre-eclampsia probabilities in these groups by 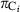 and 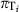, and the total number of individuals in each group by 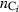 and 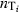. The first stage of the hierarchical model is given by

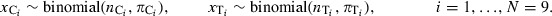


In this case the natural parameters are 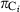 and 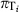 whereas the main parameter of interest, *τ*_*i*_, is the treatment effect for study *i*, defined as the log-odds-ratio for treatment compared with control as follows:

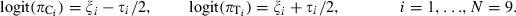


Here we wish to treat the *ξ*_*i*_s as nuisance parameters, and so we assign independent *N*(0,100^2^) priors to each. The same prior is specified for the *τ*_*i*_s in our stage 1 analysis, whereas in the full hierarchical model the *τ*_*i*_s are assumed to arise from a normal population distribution with unknown mean *μ* and standard deviation *σ*: 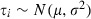, *i*=1,…,*N*. The population mean and standard deviation are assigned vague *N*(0,100^2^) and Unif(0,10) priors respectively.

#### 3.4.2. Abdominal aortic aneursym model

Let *x*_*ijk*_ denote the *k*th AAA diameter measured (at time *t*_*ijk*_) for individual *j* in study *i*. Further, let *T*_*ij*_ denote the time to rupture or to censoring for individual *j* in study *i*, and let *δ*_*ij*_ denote whether individual *j* in study *i* was censored (*δ*_*ij*_=0) or not (*δ*_*ij*_=1). The growth data are modelled as follows:

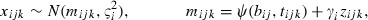
 where *z*_*ijk*_ is an indicator variable equal to 1 if *x*_*ijk*_ was measured via a computed tomography scan and 0 if it was measured via ultrasound, *γ*_*i*_ is the effect of measuring AAA diameter via computed tomography scan in study *i* (where appropriate), and *ψ*(·) is a growth curve defined in terms of parameters *b*_*ij*_ and time *t*, e.g.

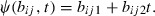
(9)

More generally, we denote the dimension of the *b*_*ij*_-vectors by *r*.

We assume that the hazard of rupture *h*(·) depends log-linearly on the underlying AAA diameter *ψ*(*b*_*ij*_,*t*), and that censoring (by surgery, non-rupture death or end of follow-up) is non-informative (Sweeting and Thompson, 2011). Then the likelihood due to the time-to rupture data is given by

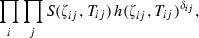
 where

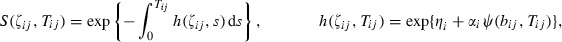
 and 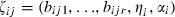. The log-linear form for *h*(·), in combination with equation (9), ensures that the hazard function is analytically integrable, allowing the likelihood to be expressed in closed form. The individual-specific parameters *b*_*ij*_ are assumed to arise from study-specific multivariate–normal population distributions:

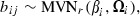
 where *β*_*i*_ denotes the study-specific mean growth parameters, which together with *η*_*i*_ and *α*_*i*_ form the study-specific parameters of interest *θ*_*i*_, and Ω_*i*_ is the interindividual covariance of growth parameters for study *i*. The goodness of fit of the study-specific models was assessed on maximum likelihood fits via the method described in Rizopoulos *et al.* (2010)—see, for example, appendix B in the on-line supplement. However, this is not the focus of our paper.

We perform three analyses, as outlined below.

- In our stage 1 analysis the initial parameters of interest 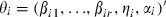 are assigned independent 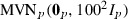 priors, where *p*=*r*+2, **0**_*p*_ is a vector of *p* 0s and *I*_*p*_ denotes the *p*×*p* identity matrix. Meanwhile, in the full hierarchical model we shall assume that 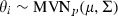, *i*=1,…,*N*, with 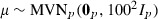. Both univariate and multivariate meta-analyses can be considered. The former requires 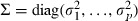, where the between-study standard deviations *σ*_*l*_, *l*=1,…,*p*, are assigned Unif(0,100) priors, for example. In the multivariate case Σ is non-diagonal with an inverse Wishart prior, say. A third alternative is to perform meta-analysis only on a subset of the parameters. Since interest primarily lies in *η*_*i*_ and *α*_*i*_ we consider a bivariate meta-analysis where the two between-study standard deviations are assigned Unif(0,100) priors and the between-study correlation parameter is assigned a Unif(−1,1) prior.
  For illustration of the above model, we restrict the growth curve to be linear as in equation (9) and define

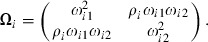
  The remaining (nuisance) parameters for study *i* are assigned the following priors, both in stage 1 and in the full hierarchical model:

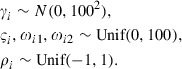
- Aside from the parameters that are defined above, particular interest also lies in calculating the probability of rupture over a short time period (*t*,*t*+*u*) given diameter *d* at time *t*. This can be approximated by

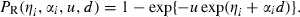
(10)
  It is straightforward to conduct a two-stage, fully Bayesian meta-analysis for this quantity. We first transform to the logistic scale for compatibility with the assumption that study-specific parameters of interest arise from a normal population distribution. After choosing values for *u* and *d* we can obtain a stage 1 posterior for the logit probability of rupture in study *i* by calculating the logit of expression (10) for each simulated value of *η*_*i*_ and *α*_*i*_. We then perform an additional stage 2 analysis, as described in Section 3.2, in which the logit probability of rupture is the only parameter of interest. Importantly, however, we are interested in a range of values for *u* and *d*, specifically in this paper the probabilities of rupture within time periods of between 3 and 24 months, in 3-month intervals, for a baseline diameter of 50 mm. Handling this situation in a one-stage framework would require repeated reparameterization of the model followed, each time, by a full analysis of all data. In contrast, our two-stage approach simply requires transformation of stage 1 output followed by a rapid second-stage analysis for each *u* and *d*.
- Another prediction of interest is the probability of crossing the surgical threshold (55 mm) over the time period (0,*u*) given diameter *d* at time 0 (baseline). This can be expressed by

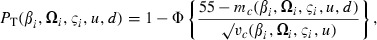
(11) where Φ denotes the standard normal cumulative distribution function, and in the linear growth case

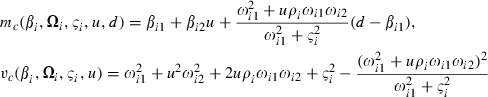
 are the model-predicted conditional mean and variance respectively of a measurement taken at time *u* given a baseline diameter *d*. This is considerably more complex than the quantity that is defined in equation (10), and we would certainly not wish to parameterize our model in terms of it, but it is meta-analysed just as straightforwardly, by first calculating the logit of expression (11) for each simulated value of *β*_*i*_, Ω_*i*_ and *ς*_*i*_ in stage 1. Again, we are interested in a range of values for *u* and *d*, as outlined above.

An assumption that the parameters of interest are drawn from some population distribution (typically normal) is a fundamental requirement of meta-analysis. This means that the underlying hierarchical model may change as different parameterizations are considered, as in (a)–(c) above, since a non-linear function of normally distributed parameters cannot, in general, also be normal. The choice of which parameters to assume normally distributed, in any given setting, is subjective. In practice, rather than assuming one parameterization to be *truly* normal, it may be preferable to think of several (or many) being *approximately* normal. Transformations such as the logit applied to expressions (10) and (11) can help to ensure that normality is a *reasonable* assumption.

### 3.5. Implementation issues

OpenBUGS code for implementing the models that were described in Section 3.4 is given in the on-line appendix. The main issue in performing a two-stage analysis is the transfer of information from stage 1 to stage 2. This is achieved by saving a full posterior sample (not just a summary) for each study-specific parameter of interest at the end of stage 1, and by then loading these samples as data in stage 2. Our posterior sample for the study-specific parameters from the full hierarchical model in stage 2 will consist entirely of values obtained during stage 1, but weighted accordingly. Hence we must ensure that a substantial sample is obtained in stage 1, not just for summarizing the stage 1 posteriors but also to compensate for the fact that there may be some conflict between the stage 1 and full hierarchical posteriors, which may render many of the values sampled in stage 1 obsolete. The increased resolution that a large sample offers, however, is offset somewhat by the need to store and subsequently to load large data files. Hence we tend to ensure that our stage 1 sample is as *efficient* as possible by saving only every *n*th value for each parameter of interest from the simulated Markov chains, where *n* is chosen so that successive values in the stored sample are essentially independent (facilities are available in BUGS that make selecting and storing such ‘thinned’ output very straightforward). Note that an increased stage 1 sample size also helps to prevent the second-stage sampler from becoming temporarily stuck near local posterior modes. In our experience, saving 10000 independent realizations for each parameter in stage 1 strikes a good balance between resolution and storage and data loading, although this may be insufficient for high dimensional problems. We are not aware of any actual restrictions on sample size and/or the number of parameters in BUGS, but larger samples will require more ‘loading’ and ‘compilation’ time—this took only a few seconds, however, for our most complex analysis (10000 samples × 14 studies × 9 parameters).

## 4. Results

### 4.1. Pre-eclampsia data

Two-stage analysis of the pre-eclampsia data was performed by using the OpenBUGS code given in the on-line appendix (appendices A.1 and A.4). The stage 1 models were run for 200000 iterations following convergence of the MCMC simulation, and the resulting samples were thinned by 20, on inspection of auto-correlation plots, to achieve approximate independence between successive values. Thus 10000 posterior realizations were generated for each study level parameter of interest *τ*_*i*_. Two analyses were conducted at stage 2: the first using just 1000 of the posterior samples from stage 1, and the second using all 10000. Both stage 2 analyses were run for 100000 iterations after convergence. A one-stage analysis was also carried out for comparison of results, again using 100000 iterations after convergence. For all analyses, the Markov chains generated were well behaved and convergence was assessed by visual inspection of ‘chain history’ plots (where a continuous line joining successive samples together is plotted against iteration number), as outlined in Lunn *et al.* (2012), section 4.4. Confirmatory analyses ran two MCMC simulations in parallel and assessed convergence formally by the method of Gelman and Rubin (Gelman and Rubin, 1992; Brooks and Gelman, 1998), an implementation of which is available in BUGS. In all cases an initial burn-in of 1000 iterations was deemed sufficient.

**Fig 1:**
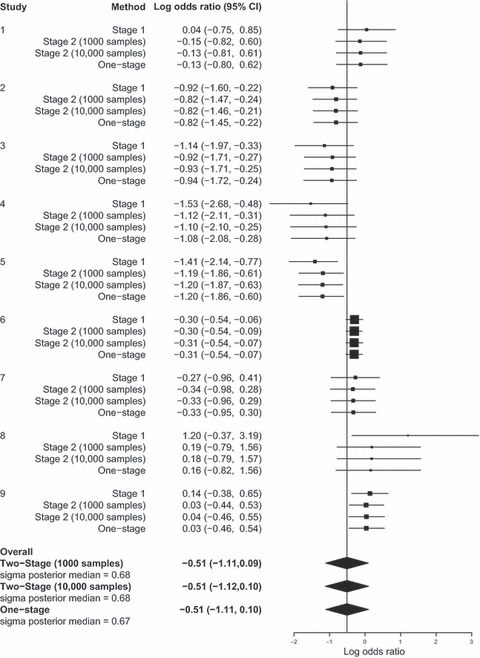
Results of the two-stage and one-stage analyses of the pre-eclampsia data: estimates are posterior medians with 95% credible intervals; the medians are shown as squares with area inversely proportional to the posterior variance; the edges of the diamonds used to denote overall estimates correspond to the limits of the 95% credible interval, whereas the central vertices show the posterior median

Our results are summarized in the forest plot shown in Fig. 1. For each study we plot the posterior median value of *τ*_*i*_ and the corresponding 95% credible interval from each of the four analyses alluded to above:

- stage 1 analysis;
- stage 2 analysis with 1000 stage 1 samples;
- stage 2 analysis with 10000 stage 1 samples;
- one-stage (simultaneous) analysis.

There is excellent agreement between posterior summaries corresponding to the full hierarchical model. Hence both stage 2 analyses perform well in terms of summarizing the study level posteriors. This is true even for studies with relatively few data (studies 4 and 8, say) where shrinkage effects in the hierarchical model are relatively strong. Also shown in Fig. 1 is a comparison of point and interval estimates for the overall mean log-odds-ratio from the three analyses corres ponding to the hierarchical model. A more complete comparison of overall inferences from these analyses is given in Table 1, where the between-study heterogeneity *σ* and the predicted effect in a new study *τ*_new_ are also considered (Higgins *et al.*, 2009). Again the agreement is excellent, confirming that our method also works well in terms of overall inferences.

**Table 1:** Comparison of two-stage analyses with 1000 and 10000 stage 1 samples to a one-stage analysis (pre-eclampsia data)†

*Parameter*	*Two-stage analysis (1000 samples)*	*Two-stage analysis (10000 samples)*	*One-stage analysis*
*μ*	−0.51 (−1.11, 0.09)	−0.51 (−1.12, 0.10)	−0.51 (−1.11, 0.10)
*σ*	0.68 (0.28, 1.56)	0.68 (0.27, 1.57)	0.67 (0.27, 1.57)
*τ*_new_	−0.51 (−2.26, 1.24)	−0.51 (−2.28, 1.26)	−0.51 (−2.25, 1.23)

† Posterior medians for overall parameters with 95% credible intervals in parentheses.

**Fig 2:**
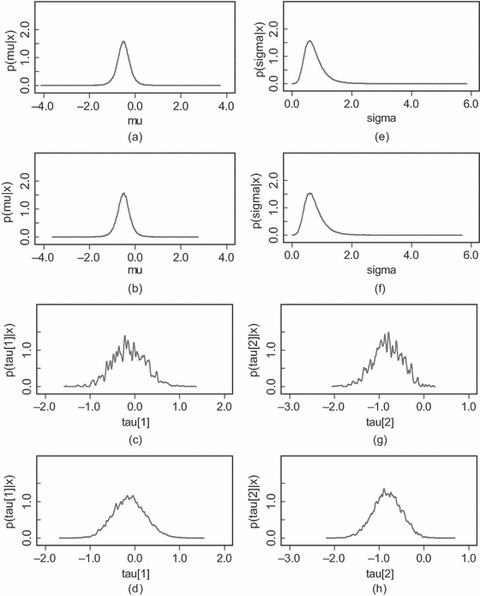
Posterior density estimates for *μ*, *σ*, *τ*_1_ and *τ*_2_ based on 100000 stage 2 samples from analysis of the pre-eclampsia data: (a) 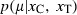 by using 1000 stage 1 samples; (b) 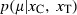 by using 10000 stage 1 samples; (c) 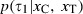 by using 1000 stage 1 samples; (d) 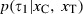 by using 10000 stage 1 samples; (e) 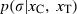 by using 1000 stage 1 samples; (f) 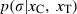 by using 10000 stage 1 samples; (g) 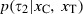 by using 1000 stage 1 samples; (h) 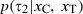 by using 10000 stage 1 samples

Fig. 2 compares posterior density estimates from the two two-stage analyses, purposely using the same bandwidth in both cases to emphasize the effect of the stage 1 posterior sample size. Inferences on the overall parameters *μ* and *σ* are virtually identical, suggesting that these are not strongly dependent on the number of stage 1 samples collected. Fig. 2 also shows density estimates for *τ*_1_ and *τ*_2_ as examples of study-specific parameters. In the cases where only 1000 stage 1 samples have been used the density estimates are somewhat ‘granular’ because there are fewer values to choose from, which reduces the resolution with which the target density can be represented.

### 4.2. Abdominal aortic aneurysm data

**Fig 3:**
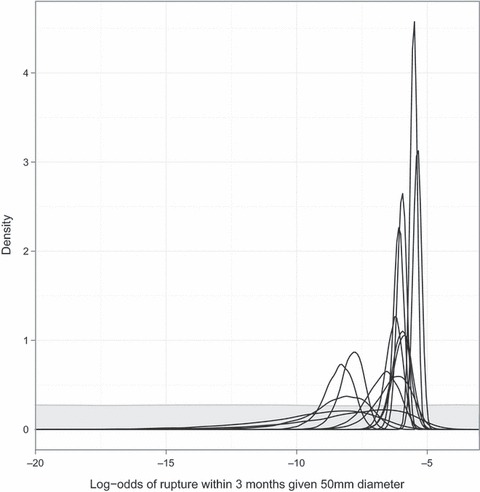
Kernel density estimates for all 14 stage 1 posteriors for the log-odds of rupture within 3 months (0.25 years) given a baseline diameter of 50 mm, logit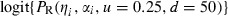, from independent analyses of study-specific AAA data: 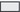, prior distribution, scaled (arbitarily) so that it is visible on the same plot

BUGS code for the stage 1 analysis is given in the on-line appendix A.5. In practice, to ensure that the parameters are less correlated, all time variables (*t*_*ijk*_ and *T*_*ij*_) are centred at the mean follow-up time for the study, whereas, in the hazard function, 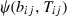 is centred at the study mean AAA diameter. Transformations are then required to obtain common parameters across the studies. Such centring is not necessary but can improve convergence substantially. For monitoring convergence, each stage 1 analysis was conducted with two MCMC chains running in parallel; the method of Gelman and Rubin (Gelman and Rubin, 1992; Brooks and Gelman, 1998) was then used. A typical analysis involved a burn-in of 6000 iterations, with 100000 further iterations thinned by 20. In all cases, sufficient iterations for obtaining 10000 approximately independent posterior realizations for each study level parameter of interest were performed. Even with the aforementioned centring to improve convergence, each stage 1 analysis took several hours to perform. Hence a single one-stage analysis of the full hierarchical model would have taken of the order of days to perform. Bearing in mind that there are numerous parameters of interest in this setting, a two-stage approach was considered essential. Prior distributions for all parameters, including derived parameters, were effectively flat within the range of values supported by the corresponding posterior, as illustrated in Fig. 3 for the log-odds of rupture within 3 months (0.25 years) given a baseline diameter of 50 mm, 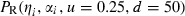.

At stage 2, to ensure comparability between studies, intercept parameters *β*_*i*1_ were standardized to represent a mean AAA diameter at *t*=0, whereas log-baseline-hazards *η*_*i*_ were standardized to represent the log-hazard at a diameter of 40 mm. Both univariate and bivariate two-stage analyses for the parameters *η*_*i*_ and *α*_*i*_ were performed. In addition, a range of univariate two-stage analyses for the derived parameters 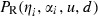 and 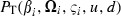 was conducted, specifically to make predictions for time periods of between 3 months and 2 years, in 3-month intervals (*u*=0.25,0.5,0.75,…,2), for individuals with a diameter of 50 mm (*d*=50). In each stage 2 analysis, 200000 realizations were generated after an initial burn-in of 10000 iterations (convergence was assessed by visual inspection of chain history plots and by Gelman and Rubin’s method in confirmatory analyses).

Table 2 shows the overall estimates for the basic parameters *η*_*i*_ and *α*_*i*_. For comparison, a classical random-effects summary estimate (DerSimonian and Laird, 1986) is also calculated by using the posterior medians and standard deviations from stage 1. Point and interval estim ates for the overall parameters are similar between the Bayesian two-stage and the classical random-effects approaches. In addition, univariate and bivariate stage 2 analyses give almost identical results. The population mean log-baseline hazard (for an AAA diameter of 40 mm), *η*, is low and signifies a median rupture rate, exp(*η*), of 1.8 (95% credible interval 1.1–2.7) per 1000 person-years. However, the hazard increases significantly with diameter, with a population median hazard ratio, exp(*α*), of 1.13 (95% credible interval 1.09–1.17) per millimetre increase in AAA diameter.

**Table 2:** Overall estimates (population medians) for main parameters of interest from Bayesian two-stage analysis of AAA data†

*Parameter*	*Estimates for Bayesian two-stage analysis*		*Classical Der Simonian and Laird (1986) estimates*
	*Univariate*	*Bivariate*	
*η*_*i*_	−6.3 (−6.8, −5.9)	−6.3 (−6.8, −5.9)	−6.3 (−6.7, −5.9)
*α*_*i*_	0.12 (0.082, 0.16)	0.12 (0.081, 0.15)	0.13 (0.092, 0.16)
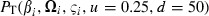	0.0015 (0.00076, 0.0026)	—	0.0018 (0.0012, 0.0027)
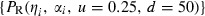	0.018 (0.0082, 0.037)	—	0.018 (0.012, 0.028)

† Classical DerSimonian and Laird (1986) estimates are also given for comparison.

**Fig 4:**
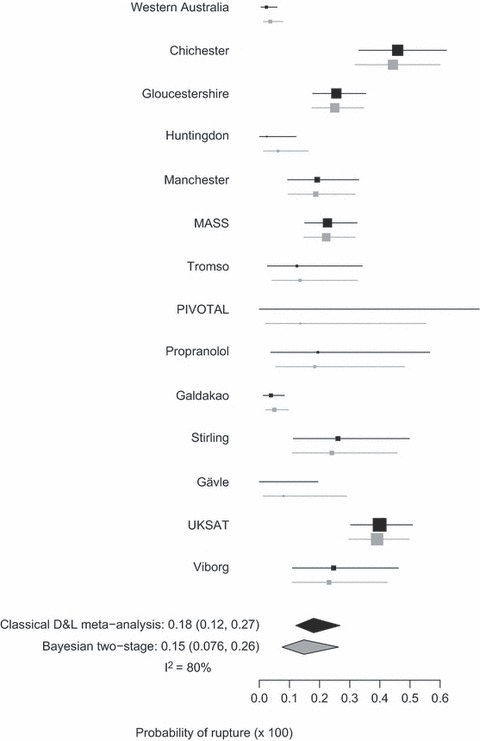
Results of stage 1 (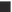, 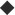) and stage 2 (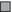, 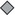) analyses of AAA data for the probability of rupture within 3 months, given 50 mm diameter: the estimates are posterior medians with 95% credible intervals; the medians are shown as squares with an area inversely proportional to the posterior variance on the logit scale; *I*^2^ is the proportion of total variation due to heterogeneity between studies; the diamond notation is for overall estimates as in Fig. 1

Currently, patients with an AAA diameter between 45 and 54 mm, identified in the National Health Service AAA screening programme, are invited back for re-screening after 3 months. To assess the appropriateness of this monitoring interval, we begin by calculating, for an individual with diameter 50 mm, the study-specific predicted probabilities of rupture and of crossing the surgical intervention threshold (55 mm) within a 3-month period, 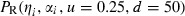 and 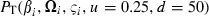 respectively. In the second stage, a hierarchical structure is placed on the logit of each predicted probability, by assuming that the study-specific values originate from a common (normal) population distribution. Table 2 shows the overall estimates transformed back to the probability scale. These now have considerably wider credible intervals than those obtained via classical random-effects meta-analysis. Results indicate that the current 3-month screening policy is relatively safe, with point estimates (and 95% credible intervals in parentheses) for the overall/population-median values of the probabilities of rupture before next screen and of crossing the intervention threshold within 3 months being 0.15% (0.076–0.26%) and 1.8% (0.82–3.7%) respectively. There is considerable between-study heterogeneity in these quantities, however, which raises the question of whether there are patient or study level characteristics that may explain this; however, this topic is not pursued here. To illustrate the level of heterogeneity, Fig. 4 shows a forest plot of the stage 1 and stage 2 posterior distributions (medians and 95% credible intervals) for the probability of rupture within 3 months, given a diameter of 50 mm. Note that study-specific estimates are variable, and that those from stage 2, corresponding to the full hierarchical model, are generally more precise than those from stage 1. Considerable shrinkage is also apparent for several studies.

**Fig 5:**
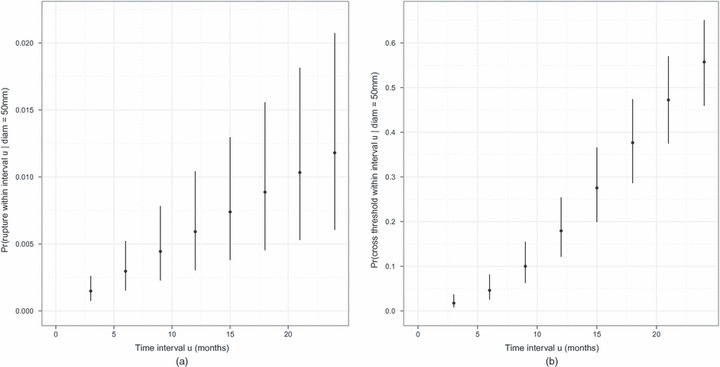
Population median probabilities of clinically significant events occurring within time periods of between 3 and 24 months, in 3-month intervals: (a) probability of rupture given a diameter of 50 mm; (b) probability of crossing the surgical intervention threshold given a diameter of 50 mm; estimates are posterior medians with 95% credible intervals

Fig. 5 shows the probabilities of rupture and of crossing the intervention threshold, given *d*=50, for various values of the monitoring interval *u*. These are obtained straightforwardly by adapting the procedure that was outlined above for *u*=0.25. We can see that monitoring intervals of 9 months or less and 6 months or less respectively are required to be confident that the population median probabilities of rupture and of crossing the intervention threshold are below 1% and 10%. However, given the degree of between-study variation, it would seem inappropriate to use this as a basis for justifying a longer monitoring interval.

## 5. Discussion

We have developed a novel method for analysing fully Bayesian hierarchical models in two stages; to the best of our knowledge this approach has not been used before. The method can be thought of as a type of particle filter (sequential Monte Carlo sampling; Doucet *et al.* (2001); Andrieu *et al.* (2010)), where the resampling is done via Metropolis–Hastings sampling; the considerable literature on particle filtering may thus point to ways of improving or extending our method. Our approach is computationally efficient and can be easily applied in settings where a one-stage analysis is difficult or impossible (e.g. for complex parameters of interest) or inefficient (e.g. when there are multiple parameter sets of interest). Although we have demonstrated, here, that the method works for a simple binomial example, we have also tested it within various settings, such as Poisson regression, and have discovered no further issues. In addition, we can see no theoretical or intuitive reason why it should fail, as long as stage 1 posteriors are obtainable. Where applicable, our approach allows potentially complex problems to be broken down into a series of more manageable problems, facilitating, for example, individually tailored analyses for each study in stage 1, and allowing a wide range of inferences to be readily obtained following a single stage 1 analysis.

In cases where the parameters of interest are complex functions, such as the probability that is associated with some clinically relevant event, a two-stage approach offers a surprisingly simple solution, avoiding the need for reparameterization of the model and subsequent reanalysis of all data. If an overall estimate of the parameter of interest is sufficient, and we require no heterogeneity measure, then another option might have been to estimate a posterior distribution for the parameter of interest by transforming simulated values for the population mean parameters *μ* from some basic analysis. However, the resulting quantity would not, in general, be meaningful, since it would not normally represent the population mean or median value, say, or any other established measure of centrality, on the scale of interest (because, for example, *f*(*E*[*x*])≠*E*[*f*(*x*)] for non-linear *f*(·)). Also, predictions on the scale of interest, for new studies, say, would not be possible by using such an approach.

One limitation of our method is the requirement for the stage 1 posteriors, obtained under an assumption of marginal independence between studies, to provide reasonable approximations to the full conditional distributions for the study-specific parameters in the hierarchical model, where studies are typically assumed *conditionally* independent instead, given the overall parameters. If there is too much conflict between these distributions then the Metropolis sampler may become degenerate, accepting only a few of the stage 1 samples. However, we would expect that this is unlikely to happen, since the most likely cause of such conflict is when shrinkage effects would be strong in the hierarchical model, owing to limited data from particular studies, in which case the corresponding stage 1 posteriors will be wider. In extreme cases, we may lose some resolution unless very large MCMC samples are taken of the posteriors from stage 1.

Another limitation occurs when data are sparse and some of the stage 1 posteriors are thus improper. Hence they cannot be obtained for use as proposal distributions. The method proposed may, at least, be used partially for such data sets, with the units for which a stage 1 posterior is available handled as described in Section 3.2, and the remaining units modelled as they would be in a standard, one-stage analysis. (In such cases it is convenient to rearrange the data so that the units for which only sparse data are available form a contiguous block.)

Our method has been presented in a meta-analysis context but is an entirely general advance, which is applicable, in principle, to a wide range of hierarchical modelling scenarios. We have implemented it in freely available software, OpenBUGS (www.openbugs.info), which is sufficiently flexible that the user may apply the methodology to almost arbitrary problem types. As mentioned above, we require that the stage 1 posteriors are obtainable. One situation in which this is not so is when the ‘borrowing of strength’ that a hierarchical model permits is essential for the identification of unit-specific (study-specific in the case of meta-analysis) parameters. This can occur in population pharmacokinetics, for example, where longitudinal drug concentration data are available for a number of individuals and we wish to make overall inferences about the concentration–time relationship. Often, in the latter stages of drug development, many patients are followed but each may give rise to only one or two concentration measurements, thus precluding independent analyses of patient-specific data.

Model criticism within the framework proposed is an area for further exploration. Standard approaches, such as traditional residual-based methods, and more advanced techniques (e.g. Rizopoulos *et al.* (2010)) are applicable to stage 1 results, since stage 1 comprises standard analyses only. Within stage 2, study level residuals and predictive distributions are readily obtained, facilitating the use of established methods for criticism, such as Bayesian *p*-values (Gelman, 2003; Gelman *et al.*, 2004). Outlying studies could be accommodated or identified by assuming a (multivariate) Student *t* population distribution for the parameters of interest (e.g. Wakefield *et al.* (1994)). The degrees of freedom could be either prespecified or estimated as part of the analysis, the latter offering a means of assessing the appropriateness of a simpler normality assumption for the population distribution instead. Efficient ways of performing cross-validation (e.g. Marshall and Spiegelhalter (2003)) and computing the deviance information criterion (Spiegelhalter *et al.*, 2002) within stage 2 are currently under investigation.

Finally, it may be possible to extend the methodology to allow two-stage Bayesian modelling in more complex evidence syntheses, such as multiparameter evidence synthesis (Ades and Sutton, 2006; Presanis *et al.*, 2011; Sweeting *et al.*, 2008), or mixed treatment comparisons (Salanti *et al.*, 2008; Lu and Ades, 2006).

## Notes

“Supporting information for ‘Fully Bayesian hierarchical modelling in two stages, with application to meta-analysis’”
