# Supplementary material for: Fully Bayesian hierarchical modelling in two stages, with application to meta-analysis
Source: J R Stat Soc Ser C Appl Stat. 2013 Aug;62(4):551–72. doi: 10.1111/rssc.12007 (PMC3814003; doi:10.1111/rssc.12007)
Supplement: Supplementary file 1 [file rssc0062-0551-sd1.pdf]

Supporting Information for "Fully Bayesian hierarchical modelling in two stages, with application to meta-analysis"

David Lunn*, Jessica Barrett, Michael Sweeting and Simon Thompson

MRC Biostatistics Unit, Cambridge, UK

Appendix A: Implementation

Details of how to set up OpenBUGS for performing two-stage analyses can be obtained by emailing the corresponding author.

A.1 Generic code for stage two

The second stage of our method is implemented in OpenBUGS using the following code, for a `p`-dimensional set of parameters of interest.

```
model {
  for (i in 1:N) {
    dummy[i] ~ dproposem(theta[i, 1:p], samples[i, 1:p, 1:L])
    theta[i, 1:p] ~ dmnorm(mu[], Sigma.inv[,])
  }
  mu[1:p] ~ dmnorm(c[], T[,])
  Sigma.inv[1:p, 1:p] ~ dwish(R[,], rho)
  Sigma[1:p, 1:p] <- inverse(Sigma.inv[,])
}
```

The `dummy[1:N]` variable is merely a 'placeholder' for the missing likelihood from stage one. It simply allows us to create a link between each `theta[i,]` and the `L` samples generated for `theta[i,]` in stage one (`samples[i, 1:p, 1:L]`). Then we may specify level 2 onwards of the hierarchical model in the standard way. (Note that in BUGS normal distributions, both multivariate (`dmnorm`) and univariate (`dnorm`), are parameterised in terms of mean and precision (inverse-variance) rather than mean and variance.) The `dproposem(.,.)` syntax acts as a flag for BUGS to utilise a specialised updating algorithm, whereby the conditional prior for `theta[i,]` at its current value, conditional on `mu` and `Sigma.inv`, is compared to the conditional prior evaluated at a randomly chosen point in `samples[i, 1:p, 1:L]`, in order to calculate the probability of moving `theta[i,]` to the randomly chosen point. Note that the values of `dummy`, `samples`, `c`, `T`, `R` and `rho` are specified in the data set. In cases where there is only one parameter of interest (`p` = 1), the following line is used instead, in combination with assumptions of *univariate* normality (`dnorm`), say, as opposed to multivariate normality (`dmnorm`).

**Address for correspondence:* David Lunn, MRC Biostatistics Unit, Institute of Public Health, University Forvie Site, Robinson Way, Cambridge CB2 0SR, UK; e-mail: david.lunn@mrc-bsu.cam.ac.uk.

```
dummy[i] ~ dpropose(theta[i], samples[i, 1:L])
```

The `dummy[]` variable must be 'observed' in order for the new mechanism to work, but its value is irrelevant. We typically specify `dummy = c(1,1,...,1)` in the data set. The mechanism is somewhat cumbersome but it works with much greater efficiency than more natural alternatives. For example, a more intuitive syntax might be

```
samples[i, 1:p, 1:L] ~ dproposem(theta[i, 1:p])
theta[i, 1:p] ~ dmnorm(mu[], Sigma.inv[,])...
```

etc. However, in this case the `samples[i, 1:p, 1:L]` matrix is considered as a multivariate stochastic quantity, which, internally in BUGS, necessitates each element being linked to every other. For large multivariate quantities this carries a substantial computational overhead, particularly when 'building' the data structures required for running the simulation. In the former approach, `samples[i, 1:p, 1:L]` is simply data and no such linking is required.

A.2 Transferring samples from stage one to stage two

Assuming the `theta[1:N, 1:p]` matrix has been monitored during stage one, we can access the required set of samples via the `coda` button on the `Sample Monitor Tool`. Two windows are created, one containing the samples themselves and another detailing which samples correspond to which elements of the monitored variable. The samples can be reformatted for incorporation into the second stage as follows. Edit the window containing the samples themselves so that a new line is created at the beginning with three numbers on it: (i) the number of studies; (ii) the number of parameters of interest; and (iii) the number of samples for each parameter. For example, we would enter '9 1 10000' for the pre-eclampsia example. Now select `Reformat CODA` from the `Particle` menu and a new window will appear containing the full sample for `theta[1:N, 1:p]` in the correct format for use with `dproposem`.

A.3 Checking flatness of implied priors

We can check for flatness of the prior distribution for any transformed parameter by simulating from the implied prior as follows. The BUGS code below is appropriate for any scalar $\theta = f(\phi)$, where ϕ is a vector of parameters $\phi_j \sim \mathrm{N}(0, 100^2)$, $j = 1, ..., p$. A sample from the prior $p(\theta)$ is obtained by simply storing the values of θ computed in each iteration; these can then be used to construct a density estimate. The final three lines of code impose the constraint $a \leq \theta \leq b$, to restrict sampling to a specific region of interest, such as values supported by the likelihood.

```
model {
  for (j in 1:p) {phi[j] ~ dnorm(0, 0.0001)}
  theta <- f(phi) # e.g. theta <- phi[1] + phi[2]*d for some d
  z <- 1
  z ~ dbern(constraint)
  constraint <- step(theta - a)*step(b - theta)
}
```

A.4 Stage-one BUGS code for pre-eclampsia model

The following BUGS code and data list is used to perform stage one of our pre-eclampsia data analysis. Note that in BUGS the binomial distribution, `dbin()`, is parameterised such that the 'probability' and 'number of trials' parameters are the first and second arguments, respectively.

```
model {
  for (i in 1:9) {
    x.C[i] ~ dbin(pi.C[i], n.C[i])
    x.T[i] ~ dbin(pi.T[i], n.T[i])
    logit(pi.C[i]) <- xi[i] - tau[i]/2
    logit(pi.T[i]) <- xi[i] + tau[i]/2
    xi[i] ~ dnorm(0, 0.0001)
    tau[i] ~ dnorm(0, 0.0001)
  }
}


list(
x.C = c(14,17,24,18,35,175,20,2,40),
n.C = c(136,134,48,40,760,1336,524,103,102),
x.T = c(14,21,14,6,12,138,15,6,65),
n.T = c(131,385,57,38,1011,1370,506,108,153))
```

A.5 Stage-one BUGS code for AAA model

```
model{

  C<-10000

  ## Longitudinal process
  for(j in 1:N){
    x[j]~dnorm(m[j],inv.varsigma2)
    m[j]<-b[id[j],1]+b[id[j],2]*t[j]+gamma*z[j]
  }

  ## Survival process
  for(i in 1:M){
    ## Mean of longitudinal process (ultrasound) at survival time T
    mT[i]<-b[id.surv[i],1]+b[id.surv[i],2]*T[i]

    ## Mean of longitudinal process (ultrasound) at survival time t=0
    m0[i]<-b[id.surv[i],1]

    ## Hazard at survival time T
    hT[i]<-exp(eta+alpha*mT[i])

    ## Hazard at time t=0
```

```
    h0[i]<-exp(eta+alpha*m0[i])

    ## Integrated hazard for individual i from 0 to survival time T[i]
    H[i]<-(hT[i]-h0[i])/(alpha*b[id.surv[i],2])

    ## Survival function
    S[i]<-exp(-H[i])

    ## Density function of survival time T[i]
    f[i]<-hT[i]*S[i]

    ## Likelihood for survival data using the ones trick
    ones[i]<-1
    ones[i]~dbern(p[i])
    p[i]<-L[i]/C
    ## Likelihood contribution for individual i
    L[i]<-pow(f[i],event[i])*pow(S[i],1-event[i])
  }

  ## Random-effects
  for(i in 1:M){
    b[i,1:2]~dmnorm(beta[1:2],inv.Omega[1:2,1:2])
  }

  ## Priors
  beta[1]~dnorm(0,1.0E-4)
  beta[2]~dnorm(0,1.0E-4)
  eta~dnorm(0,1.0E-4)
  alpha~dnorm(0,1.0E-4)
  gamma~dnorm(0,1.0E-4)
  varsigma~dunif(0,100)
  inv.varsigma2<-pow(varsigma,-2)
  inv.Omega[1:2,1:2]<-inverse(Omega[,])
  Omega[1,1]<-pow(omega[1],2)
  Omega[2,2]<-pow(omega[2],2)
  Omega[1,2]<-rho*omega[1]*omega[2]
  Omega[2,1]<-Omega[1,2]
  rho~dunif(-1,1)
  omega[1]~dunif(0,100)
  omega[2]~dunif(0,100)
}
```

Appendix B: Goodness of fit for AAA models

Figure B1 shows Kaplan-Meier estimates (with 95% confidence intervals) of the survival function of the Cox-Snell residuals, defined as the estimated cumulative risk function evaluated at the observed event times. These are obtained from each study-specific model separately. If the assumed model fits the data well then we expect the Cox-Snell residuals to have a unit exponential distribution (solid grey line). For reliability of the plots, only studies with 5 or more ruptures are shown.

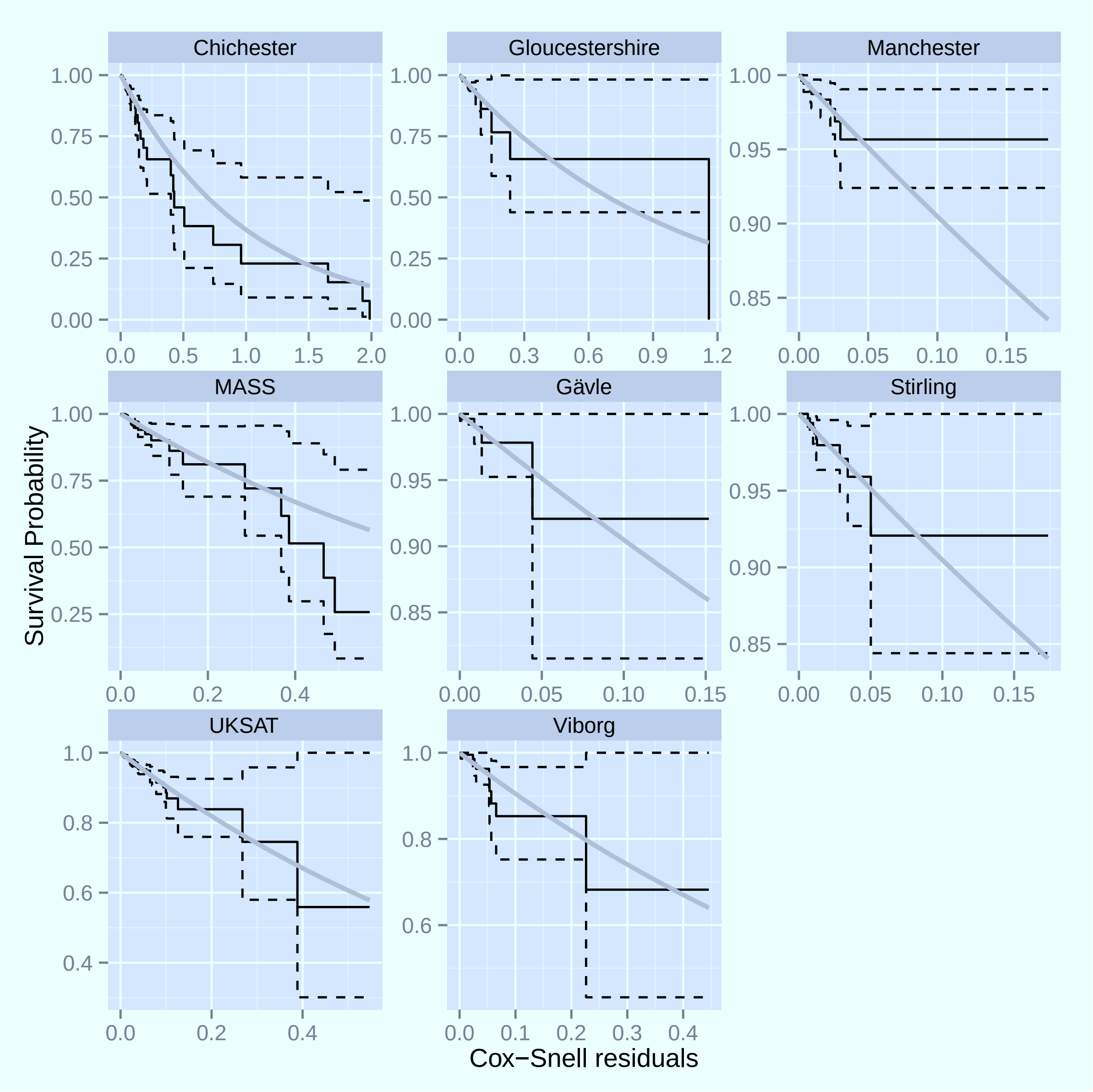

Figure B1: Kaplan-Meier estimates (black solid lines) with 95% confidence intervals (black dashed lines) of the survival function of Cox-Snell residuals. The solid grey line represents the unit exponential distribution. Only studies with 5 or more ruptures are shown.
